# Barriers and Facilitators to Acceptability of the Female Condom in Low- and Middle-Income Countries: A Systematic Review

**DOI:** 10.5334/aogh.3612

**Published:** 2022-03-10

**Authors:** Luther-King Fasehun, Sarah Lewinger, Oyinlola Fasehun, Mohamad Brooks

**Affiliations:** 1 Department of Epidemiology and Biostatistics, Temple University, US; 2 Boston University, US; 3 University College Hospital, Ibadan, NG; 4 Pathfinder International, US; 5 Boston University School of Public Health, US

## Abstract

**Background:**

Sexually transmitted infections, including HIV, remain a significant public health challenge for low- and middle-income countries, and about 111 million unintended pregnancies occur in these countries annually. The female condom is the only commonly available method that affords women and girls more control in protecting themselves from sexually transmitted infections, as well as unintended pregnancies. Yet, the female condom only accounts for 1.6% of total condom distribution worldwide.

**Objectives:**

To help fill the gaps in an understanding of what works for improved acceptability and use of the female condom in low- and middle-income countries, we conducted a systematic review of the literature that focuses on acceptability of the FC, as examined in the specific settings of intervention programs or research in low- and middle-income countries.

**Methods:**

We conducted a preliminary search of two purposively selected databases (PubMed and POPLINE) for English language articles from 2009 to 2019 with the keyword “female condom.” PubMed yielded 145 articles, while POPLINE yielded 164 articles. Included studies involve a purposive, interventional deployment of the female condom; have occurred in a low- or middle-income country, as defined by the World Bank; and have focused on acceptability of the female condom. Upon review of duplicates and abstracts, a total of 14 articles made the final selection.

**Findings:**

The included articles represent seven different countries: the Dominican Republic, El Salvador, China, Malaysia, Nicaragua, South Africa, and Uganda. We identified four key barriers to FC acceptability, including partner acceptability, functionality, aesthetics, and access. We identified four key facilitators to FC acceptability, including repeated use, supportive attitudes, protection confidence, and reproductive control.

**Conclusion:**

Effective promotion and uptake of the female condom in low- and middle-income countries can be realized if novel strategies and approaches are implemented to tackle persistent barriers to acceptability.

## Background

Unintended pregnancies and sexually transmitted infections (STIs), including human immunodeficiency virus (HIV), continue to present significant public health and socioeconomic challenges for women and girls in low- and middle-income countries (LMICs) of the world [1]. In sub-Saharan Africa, an estimated 4 200 adolescent girls and young women get infected with HIV every week [2], and 111 million unintended pregnancies occur every year across LMICs [1]. Women and girls are more vulnerable to STIs such as gonorrhea and chlamydia, which can result in pelvic inflammatory disease, ectopic pregnancy, and infertility [3]. Specific to HIV, a complex web of biological, socioeconomic, cultural, and political factors contribute to the vulnerabilities that women and girls face [4,5,6,7]. There is a growing body of evidence illustrating the socioeconomic benefits of access to modern contraception [8,9]. When women and girls can reliably obtain contraception, they can delay pregnancy and plan their family size, which can help them achieve their personal goals, while advancing their socioeconomic statuses. The ability to plan whether and when to start a family is linked to women’s increased workforce participation and greater satisfaction in relationships [10]. Unfortunately, the COVID-19 pandemic has only made matters worse, especially in LMICs, as sexual and reproductive health services and programs have seen varying dips in demand, coverage, quality, and resource prioritization [11].

The female condom (FC) is widely regarded as the only commonly available method that affords women and girls more control in protecting themselves from STIs (including HIV) as well as unintended pregnancies [12]. The FC can also be used by men who have sex with men (MSM), another group that is highly vulnerable to STIs due to biological and behavioral factors [13]. In some countries, there has been wide-ranging advocacy for the expansion of the utility of the FC, in order to accommodate, for example, anal sex. In late 2018, the U.S. Food and Drug Administration (USFDA) approved a reclassification, and the FC is now known as the “single-use internal condom” in the US [14]. Currently, the FC is the only method for preventing STIs and unintended pregnancies, that is initiated by the receiving partner [15]. There have been calls from public health practitioners and global health advocates for the inclusion of the FC in expanded sexual and reproductive health programs, especially in LMICs [12]. With support from international partners, the FC has been introduced in over 90 countries, through various mechanisms, including no-cost public distribution, commercial retailers, and social marketing [16]. Despite these efforts, the FC only accounts for 1.6% of total condom distribution worldwide [17].

There are multiple factors that could potentially influence the use of any barrier method of sexual protection such as the FC. Acceptability, in general terms, has been defined as “the quality or state of meeting one’s needs adequately” [18] and “the degree to which somebody agrees that something is good enough to use or allow.” [19] The acceptability of the FC to a user refers to the functional characteristics of the FC, along with general beliefs and perceptions about the FC, that would make the FC able to meet the user’s needs, wants, or desires, in a manner that the user considers adequate or good enough.

## Objectives

To help fill the gaps in an understanding of what works for improved acceptability and use of the FC in LMICs, given the clear need for FCs in these countries, we conducted a systematic review of the literature that focuses on user acceptability of the FC, as examined in the specific settings of intervention programs or research in LMICs. By limiting our review to articles published in peer-reviewed journals within the years 2009 till 2019, and based only on FC interventions in LMICs, we aim to establish an understanding of the recent trends, contextual factors, and key stakeholders that affect the facilitators and barriers to the improvement of FC acceptability in LMICs.

We hope this understanding will lead to improved programming, promotion, and research, in order to advance the acceptability and usage of the FC, by female and male users in countries with a high burden of HIV and other STIs, as well as countries with high total fertility rates. The global health community continues to grapple with competing priorities, including the ever-present health threats of climate change; the as-yet ongoing COVID-19 pandemic, and the threats of future pandemics; and weak and sub-optimal health systems. All of these challenges are particularly more dire in LMICs. It is hoped that, based on a stronger and more recent evidence of what works to improve acceptability and use of the FC in LMICs, this dual-purpose sexual and reproductive health commodity can be leveraged and scaled up, in order to contribute towards the mitigation of STIs and unmet need for contraception in LMICs, in light of other health and development challenges that LMICs face.

## Methods

### Selection of Published Articles

We systematically reviewed the literature in search of descriptions or evaluations of programmatic or research interventions that involved the acceptability of the FC in LMICs. To be considered in our analysis, the study had to: (1) involve a purposive, interventional deployment of the FC to a specifically described study population or sub-population; (2) have occurred in an LMIC, as defined by the World Bank [*https://datahelpdesk.worldbank.org/knowledgebase/articles/906519-world-bank-country-and-lending-groups*]; (3) have focused on acceptability of the FC; (4) have been published in English; and (5) have been published between the years 2009 and 2019, inclusive. Our literature review focused on intervention studies due to the unique advantages that such study methodology offers. Intervention studies provide detailed information about FC acceptance from individuals who have used the device. In many of the selected intervention studies, participants receive an in-depth FC training before they are given FCs to use with their partners, which helps minimize the potential for user error. Further, intervention studies contain detailed insights about FC acceptance, including users’ informed opinions about its aesthetics, design, effectiveness, sensation, and their overall level of satisfaction.

We chose the specific time period to ensure current relevance of the systematic review to stakeholders involved in sexual and reproductive health and rights (SRHR), as well as the scholarly community interested, specifically, in the FC. The second-generation FC (FC2), made of synthetic latex, was approved by the USFDA in March 2009. Following FC2’s introduction in 2009, it was approximately 30% cheaper than the first-generation FC (FC1), which was made of polyurethane, and had been approved by the USFDA in 1993 [20]. Currently, FC2 is the only USFDA-approved FC on the market [21].

### Search Strategy

**Figure 1:**
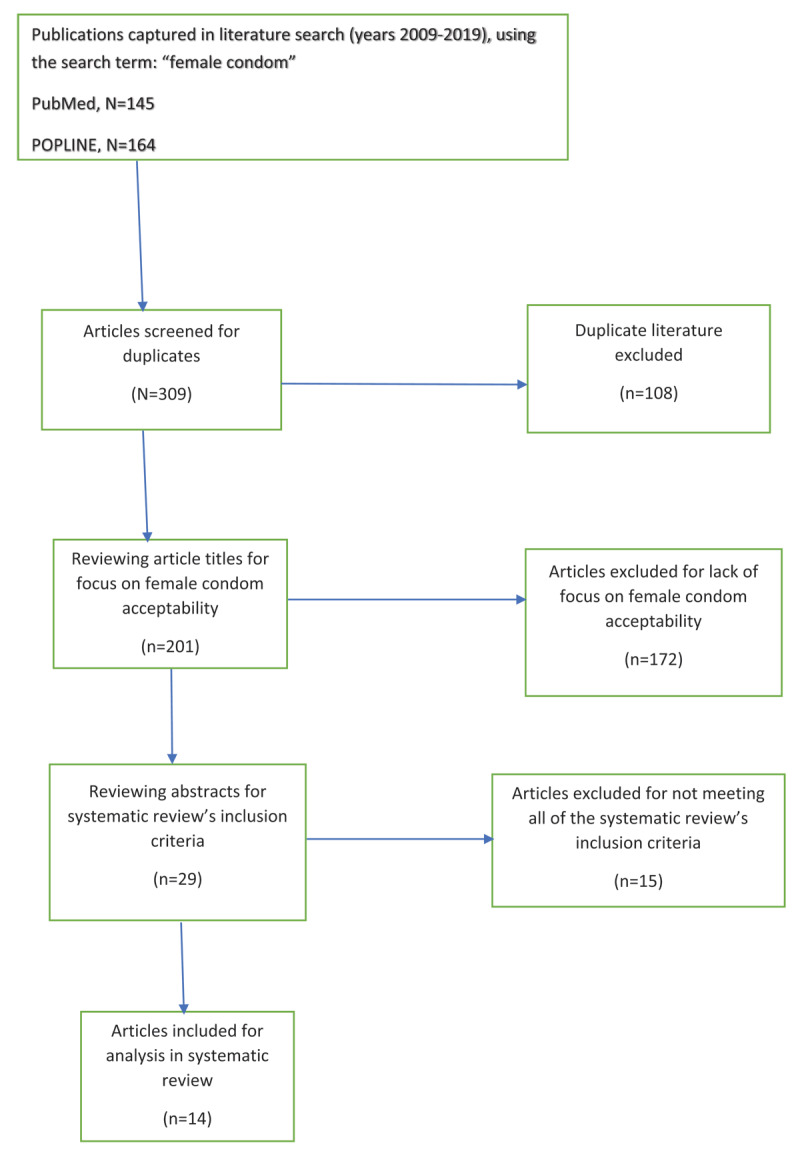
Article selection process flowchart.

We conducted a preliminary search of two purposively selected databases (PubMed and POPLINE) for English language articles from 2009 to 2019 with the keyword “female condom.” PubMed yielded 145 total results, while POPLINE yielded 164 total results. There were 108 duplicate articles. After reviewing titles to discern which articles focused on FC acceptability, we found 29 relevant articles. We then reviewed abstracts and found 14 articles that met our inclusion criteria (***Figure 1***). The study types we examined ranged from cohort studies and clinical trials to qualitative and mixed-method study designs, and these allowed for a rigorous approach where study authors could assess the effects of their respective interventions with a great deal of accuracy and precision.

### Data Extraction

We extracted details of the intervention, including design characteristics (study type, study location); specific type(s) of FC deployed; aims of the research or programme; population characteristics (population size, sample size, demographic/occupational details); year of publication; and research/programme outcomes (identified barriers to FC acceptability, identified facilitators to FC acceptability).

### Data Synthesis

For reporting purposes, we identified and separated *barriers* of FC acceptability from *facilitators* of FC acceptability. In order to facilitate interpretation of the various studies, we defined *barriers* as a group that include partner acceptability, functionality, aesthetics, and access. Further, we defined *facilitators* as a group that include repeated use, supportive attitudes, protection confidence, and reproductive control.

### Ethics Statement

This study did not involve human subject research. As such, we did not seek institutional review board (IRB) approval.

## Findings

In total, we found 14 articles that fit our inclusion criteria (***Table 1***). Geographically, the included articles represent seven different countries: China, the Dominican Republic, El Salvador, Malaysia, Nicaragua, South Africa, and Uganda. Several articles focus on high-risk demographics, such as commercial sex workers, while others focus on lower-risk populations, such as monogamous couples. This mix helps ensure the generalizability of the findings from this systematic review.

**Table 1:** Details of final sample of articles describing facilitators and barriers to female condom acceptability in low- and middle-income countries, *N* = 14.


AUTHOR	TITLE	YEAR	COUNTRY	POPULATION	METHODOLOGY	KEY BARRIERS	KEY FACILITATORS

Zhou *et al*. [22]	Short-term acceptability of female condom use among low-fee female sex workers in China: A follow-up study	2019	China	Female sex workers aged 18–60 years, who charge less than or equal to 80 RMB (approximately $0.16 USD) per vaginal sex transaction	Quantitative; follow-up study; n = 312	Partner acceptability, Functionality	Supportive attitudes

Ting, Wong, & Tnay [23]	A pilot study on the functional performance of the acceptability of an innovative female condom (Wondaleaf) in Malaysia	2018	Malaysia	Sexually active heterosexual women aged 18–50 years	Mixed methods; follow-up study; n = 51	Partner acceptability, functionality	Reproductive control, protection confidence

Schuyler *et al*. [62]	Building young women’s knowledge and skills in female condom use: Lessons learned from a South African intervention	2016	South Africa	Black South African female full-time university students, aged 18 years and older, HIV-negative, self-reported condom-less vaginal intercourse in the previous 2 months, not pregnant or wanting to be pregnant in the next 9 months	Mixed methods; randomized controlled trial, n = 296; qualitative in-depth interviews, n = 39	Partner acceptability	Reproductive control, protection confidence

Wang, Liu, & Cheng [25]	Acceptability of the Phoenurse female condom and second-generation Femidom female condom in Chinese women	2016	China	Sexually active women aged 20-49 years, in a monogamous partnership	Quantitative; randomized, crossover clinical trial; n = 290	Aesthetics, functionality, partner acceptability	Repeated use

Wu *et al*. [31]	Short-term acceptability of the Woman’s Condom among married couples in Shanghai	2016	China	Heterosexual monogamous couples, aged 18 years and older who had been together for at least 6 months, not pregnant or breastfeeding or seeking to be pregnant, using non-barrier contraception or not at risk of pregnancy	Mixed methods; non-randomized clinical trial; n = 60 (couples)	Aesthetics, functionality	Repeated use

Mantell *et al*. [29]	Promoting female condom use among female university students in KwaZulu-Natal, South Africa: Results of a randomized behavioral trial	2015	South Africa	Full-time female university students, aged 18 years and older, HIV-negative or status unknown, not pregnant or wanting to be pregnant in the next 9 months, reported condom-less vaginal intercourse in the past 2 months	Quantitative; randomized behavioral trial; n = 296	Partner acceptability	Repeated use, reproductive control

Wang *et al*. [27]	Awareness of female condoms and failures reported with two different types in China	2015	China	Sexually active women aged 20-49 years old, in monogamous partnerships	Quantitative; randomized, crossover trial; n = 290	Access	Repeated use

Masvawure *et al*. [24]	“It’s a different condom, let’s see how it works”: Young men’s reactions to and experiences of female condom use during an intervention trial in South Africa	2014	South Africa	Sexually active men aged 18-28 years old, whose partners (female university students) were enrolled in an FC intervention trial	Qualitative; cross-sectional survey; n = 38	Aesthetics, functionality	Protection confidence

Nie *et al*. [34]	Promoting female condoms in the sex industry in 4 towns of Southern China: Context matters	2013	China	Women aged 16 years and older, sexually active in the prior 30 days	Quantitative; cohort study; n = 445	Access	Protection confidence

Liao *et al*. [33]	Female condom use in the rural sex industry in China: analysis of users and non-users at post-intervention surveys	2011	China	Women working in commercial sex establishments, aged 16 years and older, who self-reported having been sexually active in the previous 30 days	Quantitative; cohort study; n = 152 surveys	Functionality, partner acceptability, access	Supportive attitudes

Wanyenze *et al*. [26]	The new female condom (FC2) in Uganda: perceptions and experiences of users and their sexual partners	2011	Uganda	Mixed (female sex workers, sexually active women, HIV+ men and women, health providers)	Qualitative; 16 in-depth interviews (8 women and 8 men); 8 focus group discussions (women); 22 key informant interviews (providers)	Partner acceptability, access	Supportive attitudes, reproductive control

Hou *et al*. [28]	A crossover comparison of two types of female condoms	2010	China	Female sex workers	Quantitative; crossover randomized clinical trial; n = 291	Functionality, partner acceptability, aesthetics	Supportive attitudes

Mack *et al*. [30]	Introducing female condoms to female sex workers in Central America	2010	El Salvador and Nicaragua	Female sex workers	Mixed methods; cross-over trial; two rounds of FGDs (n = 115 first round, n = 81 second round); IDIs with individual FSWs	Functionality, access	Repeated use, supportive attitudes, reproductive control

Lara *et al*. [32]	Acceptability and use of the female condom and diaphragm among sex workers in Dominican Republic: Results from a prospective study	2009	Dominican Republic	Female sex workers, aged 18-35, not currently pregnant or wanting to be pregnant in the next 5 months, having at least four instances of vaginal sex in the last month	Quantitative; prospective survey; n = 243	Functionality	Supportive attitudes, repeated use

Superscript numbers on the names of author(s) refer to the References in main manuscript							


### Barriers

We identified four key barriers to FC acceptability, including partner acceptability, functionality, aesthetics, and access.

#### Partner Acceptability

Across various studies set in different LMICs, we see how low or absent partner acceptability serves as a barrier to FC acceptability and use. Zhou *et al* conducted a follow-up study over one month to assess the acceptability of the FC among female sex workers in China [22]. Over the course of the study, and of a total 312 female sex workers that were enrolled in the study, only 273 reported using FCs, and 50.3% (n = 157) reported that they would not use the FC in the future. While there were various reasons for unwillingness to use the FC again, 50 out of the 157 (31.8%) reported that their clients refused to use FCs. In Ting *et al*’s study of the Wondaleaf FC in Malaysia [23], 29.4% (n = 15) of participants dropped out of the study due to male partner disapproval. Here, the study authors found that male partner acceptance was the best predictor for future use of the device, arguing that patriarchal beliefs around sex and gender are major obstacles to FC uptake. In South Africa, a survey of men whose female partners were enrolled in an FC intervention trial found that many of the men felt “powerless” after using the FC, because it transferred contraception’s control from men to women [24].

In a randomized clinical trial evaluation of the acceptability of the Phoenurse FC and second-generation Femidom (FC2) among women in monogamous partnerships at a family planning clinic in China [25], 91 (31.7%) of the 290 participants reported that their male partners refused to use the FC, due to discomfort (n = 39), unpleasant noises (n = 21), dissatisfaction with FC appearance (n = 15), impact on sexual pleasure and performance (n = 5), and unspecified reasons (n = 8). This illustrates the range of reasons why male partners may refuse to use the FC. In Uganda, a qualitative methods approach (16 in-depth interviews and 8 focus group discussions) assessed the acceptability of the FC2 by women and their male partners [26], Here, the majority of female participants reported having to negotiate with their partners before sex, in order to use the FC2, and this was mainly due to initial male partner resistance. However, most women reported that their male partners liked the FC2 once they tried it.

#### Functionality

The primary function of the FC is to serve as an effective physical barrier that prevents contact of genitals and their secretions (including ejaculated semen) during sexual intercourse. Across studies in LMICs, a range of assessments of the functionality of FCs is seen. In one study, only 4 clinical failures (2.60%) out of the total 155 uses of the Wondaleaf FC was reported [23]. However, several users reported partial or perceived slippage: (i) The feeling that the Wondaleaf FC was slipping inside their vagina, even though it was not slipping; (ii) The occasional slipping of the Wondaleaf FC’s pouch, which could be reinserted; (iii) The slipping of the pouch, which could not be reinserted. While none of these were considered complete slippage, the third type of slippage was included as one of the clinical failures, since the user was potentially exposed to bodily fluid exchange. In total, 24 partial slippage incidents (15.48%) were reported out of the 155 Wondaleaf FC uses.

Similar slippage incidents have been reported in other studies, including one of FC2 acceptability among female sex workers and HIV-positive and HIV-unknown men and women in Uganda [26]. Here, female sex workers reported needing to hold the FC2 so that it stayed in place. Also, 2 of 8 total male participants reported that the FC2 required more care than the male condom during intercourse, and their sexual performance was affected due to the fear of slippage. In a randomized crossover trial in China, slippage was the most prevalent type of clinical failure, affecting 6.9% of Phoenurse FC uses and 5.0% of FC2 uses [27]. In another study of the Phoenurse FC, slippage was found to be widespread, with complete or partial slippage reported for 10.9% of Phoenurse uses and 9.1% of FC2 uses [28].

In addition to slippage, insertion has been shown to be challenging for FC users. In a study of the short-term acceptability of the Woman’s Condom (a slight modification of the FC which possesses a dissolving polyvinyl alcohol capsule, to aid insertion) among married couples in China [29], of all 60 couples trained on insertion, 11 women reported that they did not know how deeply to insert the device, and if the device was correctly inserted; 6 women reported difficulty inserting the device; and 4 women reported experiencing discomfort inserting the device. Insertion was the most common challenge reported by South African men who used the device with their female partners [24]. In an FC intervention with female sex workers in El Salvador and Nicaragua, difficulty to insert and remove the FC, as well as physical discomfort associated with the internal ring during insertion, was prominent [30].

#### Aesthetics

The appearance and size of the FC were frequently reported as reasons for non-acceptance of the device. In a study of the Woman’s Condom in China, appearance was cited as the most disliked feature, along with insertion difficulty [31]. However, studied women preferred the WC’s appearance to several other types of FC, including the FC2 and V-Amour. Another study from China showed that 9.3% of participants cited an unappealing appearance as reason for non-acceptance of the FC [28]. It was further noted that users significantly preferred the aesthetics of the Phoenurse to the FC2, with 22.7% (n = 66) of participants reporting that the FC2’s appearance/design was unsatisfactory, while only 13.1% (n = 38) found the PFC’s appearance/design unsatisfactory.

Focus group discussions with female sex workers in El Salvador and Nicaragua elicited negative responses to the size of the FC, both due to the fear that the large size would cause insertion difficulty, and that it would be hard to hide or conceal the device [30]. Due to the highly stigmatized nature of sex work, being able to discreetly carry the device is an important consideration for this high-risk demographic. Similar concerns for discreetness were expressed in South Africa, among men, regardless of whether their partners were sex workers or not [24].

#### Access

Several studies found that lack of access is a serious obstacle to widespread adoption of the FC. In China, individuals can easily obtain male condoms at supermarkets, pharmacies, and hospitals, but those same venues rarely stock FCs [27]. Access was a persistent obstacle to FC use among female sex workers in El Salvador and Nicaragua, due to the limited availability of the device in places where male condoms are sold, and the potential unsubsidized retail price of 2–3 US$ per FC [30].

### Facilitators

We identified four key facilitators to FC acceptability, including repeated use, supportive attitudes, protection confidence, and reproductive control.

#### Repeated Use

In a five-month follow-up study of female sex workers in the Dominican Republic, functionality issues such as the penis slipping between the FC and vagina became increasingly rare as participants developed experience with the method [32]. A survey of 290 sexually active women in monogamous relationships was completed in China, dividing the women into two groups to test the acceptability of the FC2 and Phoenurse [27]. Group A used 10 Phoenurse female condoms, then 10 FC2s, while Group B used 10 FC2s, then 10 Phoenurses. For both groups, all types of clinical failure decreased with repeated use, including breakage, invagination, misdirection, and slippage. Another study among heterosexual couples in China using the Woman’s Condom [31] showed that couples’ abilities to successfully use the FC generally improve after several uses. This trend is also seen among female sex workers in Nicaragua and El Salvador, with most of them stating they needed to try the FC 3 to 10 times before deciding whether they liked the device [30]. While many found it difficult to use at first, nearly every participant said it was easy to insert, and comfortable to use, once they had enough practice.

#### Supportive Attitudes

Many of the studies demonstrate that once potential users have tried the FC, they feel positively about it, and report willingness to use it again. This was especially true for older female sex workers, as well as sex workers with older clients [22,32,33]. In a study in China [22], low-fee female sex workers were more likely to accept the device, and these workers tend to have older clients with a higher prevalence of erectile dysfunction. Unlike the male condom, the FC does not require an erect penis to function. Among female sex workers in the Dominican Republic, 54% of the workers reported liking the FC, especially those above the age of 25 years [32]. Among female sex workers in two rural towns in China, most of the FC users were slightly older, married, and had families to support [33].

Sex workers in Nicaragua and El Salvador appreciated the amount of lubrication on the FC [30]. Participants noted that the male condom is drier, which can lead to urinary tract infections and irritation. These supportive attitudes about the amount of lubrication was also seen in studies in Uganda [26].

#### Protection Confidence

In numerous studies, participants expressed confidence in the ability of the FC to prevent unwanted pregnancies and STIs. In a study of the Wondaleaf FC in Malaysia, most participants affirmed that the device protected against pregnancy and STIs [23]. Among female sex workers in China [34], FC users tended to use the device to prevent HIV infection. Studies among female South African university students also demonstrated a protection confidence with FC use, along with greater intention to use FC in the future [29]. Protection confidence is also used to negotiate FC use [24]. In El Salvador and Nicaragua, many sex workers believed the FC offered better protection against pregnancy and STIs than the male condom [30], including better protection for the labia.

#### Reproductive Control

Several studies found that women appreciated the greater sense of reproductive control that the FC afforded them. In a study in Malaysia, most participants believed that the FC promoted a greater sense of control over their health and personal hygiene [23]. Female sex workers in El Salvador and Nicaragua appreciated being able to insert the device prior to the initiation of sexual activity [30]. One participant explained that “[We] can put it in ahead of time. For example, if a client arrives and we already know that he does not like to use a condom, we can go to the bathroom a half-hour ahead of time, and put it on.” With the FC, they can protect themselves even with male partners who refuse to use male condoms. Compared to the male condom, there seems to be less concern among women about their male partners tampering with the FC, or removing the FC during sexual intercourse.

## Discussion

Decades after the introduction of the FC, and despite concerted efforts by the global health community to promote uptake of the only available method for preventing STIs and unintended pregnancies, as initiated by the receiving partner, the FC only accounts for less than 2% of total condom distribution worldwide [17]. In nationally representative household surveys that looked at FC practices and behaviors, ever-use of FC among women of reproductive age was less than 1% in most developing countries, with the exception of Zambia, Malawi, Guyana, Swaziland, and South Africa, which ranged from 1–7% [35]. The results of our systematic review shed more light on the many facets of this underutilization challenge, and provide the basis for recommendations to improve acceptability and use of the FC for improved sexual and reproductive health in LMICs.

The proportion of study participants (as reported by the authors of the articles included in our systematic review) who noted partner acceptability, or the lack thereof, as an issue that could constitute a barrier to FC uptake ranged from *27.1%* to *greater than 50%*. While the goal of the FC is to give women greater control over their protection, studies have shown that partner acceptance and cooperation is critical for successful use of the FC [36,37,38]. Other studies have documented men’s belief that the FC gives women too much power and control over sex [39,40]. Further, functionality is another key element. Past studies have consistently shown insertion difficulties for FC users, with difficulties in insertion in as many as half of users in some studies [41,42,43,44]. Difficulties in insertion have been associated with less consistent use of the FC in other studies [45,46]. It is important to situate FC insertion difficulties within the intimacy context of sexual intercourse. Evidence shows how seemingly awkward or difficult moments in the lead up to sexual intercourse, or in the act itself, can discourage further acts of sexual intercourse [47] and even strain spousal relationships. To this end, it appears that our findings corroborate earlier findings that emphasize the importance for FC manufacturers, policy makers, and reproductive health program managers to improve training and other interventions that can aid user experience of the FC, in order to minimize the occurrence of difficulties.

Aesthetics also plays an important role in FC acceptability. Studies have shown that the appearance and large size of the FC have led to negative impressions and were often cited as reasons for rejection by male partners [48,49,50]. Our findings in this review show how aesthetics are important to both women and men, for similar or different reasons. For example, the relatively larger size of the FC, compared to the male condom, was found to be disturbing in two studies we reviewed. However, while some men found the wide spread of the FC on their female partner’s vulva area as less erotically stimulating, women generally accepted the FC’s protective ability to cover their labia, and reduce contact surface area, especially in the context of transactional sex. Also, access is a critical factor determining the acceptability, or lack thereof, of the FC. Past studies have highlighted that the high cost of the FC, in comparison to the male condom, has resulted in limited access, promotion, and sustained use of the method [16,41]. A negotiated price point of 0.57 US$ per FC was set for developing countries between UNAIDS and manufacturers; however, this is significantly more expensive than the cost of the male condom [16]. In our review, 5 out of the 14 studies we analyzed described poor or absent access as an important barrier to FC acceptability and use.

Despite these barriers, there are several key factors that facilitate the acceptability of the FC that have been noted in the literature. Repeated use has been shown to be a facilitator. Past studies have demonstrated that most users overcome initial difficulties associated with appearance, insertion and/or removal, and technical issues during sex, with practice and repeated use, and these have resulted in higher acceptability of the FC [44,38,51,52]. Supportive attitudes have also been shown to play an important role in the acceptability of the FC. Studies have shown that positive perceptions related to lack of male responsibility and enhanced sexual pleasure, both from stimulation from the external ring and lubrication, were cited as key advantages to the FC [44,52,53,54]. In addition, protection confidence was another factor highlighted in the literature. Past studies have shown that protection from STIs, including HIV, and unintended pregnancies were commonly cited as an advantage of the FC both by women in the general population and female sex workers [53,55]. Lastly, greater reproductive control was cited as a facilitator of FC acceptability among the articles assessed as part of this systematic review. Several studies note that the FC has been used as a protective tool that can help address gender inequalities and power imbalances, especially in scenarios where partners refuse male condom use [48,56,57,58]. Overall, we see common themes across LMICs that affect acceptability and use of the FC. We strongly believe that this presents the opportunity for all stakeholders in sexual and reproductive health care, programming, and policy to better situate and operationalize their efforts at mitigating the burden of STIs (including HIV) and unintended pregnancies in LMICs.

### Recommendations

Effective promotion and uptake of the FC in LMICs can be realized if novel strategies and approaches are implemented to tackle persistent barriers to acceptability. Contexts will vary across and within countries, and the acceptability of the FC to persons differing across sociocultural, economic, and demographic factors (such as age, education, relationship status, income, religion, and sexual orientation) will be most improved if such factors are considered in the development and deployment of FC interventions. Innovative approaches for developing novel solutions, such as human-centered design (HCD) which is based on an intentional engagement of would-be end-users, can be utilized to capture insights from women and men, across sociocultural, economic, and demographic groups, to improve the design and desirability of FCs. For example, the “Panty Condom” is an innovative design that combines the FC with lingerie, and has been shown to be highly acceptable from pilot studies [59]. Other approaches to improving the aesthetics of FCs include availability of a varied mix of shapes, contours, colors, and scents. Such wide varieties, even for the same brands of FCs, can be powerful in stimulating acceptability.

The prevention of STIs and unintended pregnancies are the primary functional aims of FCs. Other functions like increased sensation and pleasure are usually considered secondary. We recommend that FC manufacturers, as well as intervention programs and policies, carefully assess their manufacturing processes, to avoid an imbalance in a potential trade-off between pleasure and safety. The use of coloring and scents can potentially affect the tensile strength of latex materials. Beyond the material from which FCs are made, we also recommend the use of digital communications solutions to link comprehensive FC instructions and tutorials with end-users. For example, in settings with high mobile phone usage (as we are seeing in LMICs), the use of QR codes in packaging that links to videos of FC demonstrations and insertion techniques could pose a great potential in improving acceptability.

Targeting men in FC programming must be intentionally scaled up, involving both a demand-side and a supply-side component to the programming. Successful FC programs are those that involve information and demonstration campaigns that target both women and men, such as Pathfinder International’s FC program in Mozambique [60]. More nuanced research is recommended in order to understand the behavioral modalities that drive male acceptance of the FC, for heterosexual and same-sex relations. We recommend social and behavior change (SBC) programs in sexual and reproductive health and rights to consider expanding the “prestige” inherent in the act of male partners initiating the use of FCs, including through private procurement. Men should feel proud to suggest FCs to their partners – this needs to be conveyed as “manly” and “ego-boosting” for males. Other communication efforts can include building capacity among women, and men who have sex with men, to effectively negotiate with male partners, and this can be incorporated as part of every FC program, through primary healthcare facilities, community health workers, and voluntary counselling and testing centers for HIV and other STIs.

Manufacturing and procurement agencies should explore the economies of scale that are present when large amounts of FCs are produced. For this to succeed, we recommend that the global health community, including multilateral institutions (e.g., the WHO, World Bank, UNFPA), national governments, bilateral aid organizations (e.g., NORAD, USAID), donor/philanthropic foundations, and the private sector actively support advanced purchase orders of FCs. We recommend the provision of free FCs to targeted populations that have high levels of acceptability, such as female sex workers.

### Strengths and Limitations

Our systematic review is possibly the first to examine LMICs as a group of countries (not limited to a geographical region, e.g., sub-Saharan Africa) when assessing factors associated with acceptability and use of the FC. However, these results all come from intervention studies which may not reflect the profile of the general population. A recent literature review of FC use in sub-Saharan African countries argued that non-intervention studies did not provide users an opportunity to experience the use of FCs firsthand, and therefore tended to rate overall acceptability negatively, due to preconceived ideas and beliefs about the FC [61]. While our systematic review addresses this concern, we also note that our results reflect the perspectives and experiences only of those who have actually used the FC.

Given the potential use of the FC as an internal condom by men who have sex with men (MSM), it is necessary to critically study FC use among this demographic. MSM are also at risk of STIs, including HIV. However, the behavioral and biological factors that affect acceptability of the FC in heterosexual intercourse is expectedly different in the context of MSM relationships. Further, due to the stigma against MSM in some LMICs, there is a critical lack of research on acceptability and usage of the FC among that demographic group. As such, our systematic review did not appraise acceptability of the FC within this important group of persons, due to lack of prior research in the area. This further limits the generalizability of our findings.
